# Anomalous origin of the right coronary artery from the mid-left anterior descending coronary artery: association with acute myocardial infarction

**DOI:** 10.1259/bjrcr.20170031

**Published:** 2017-09-07

**Authors:** Semaan Kobrossi, Charbel Saade, Wissam Alajaji, Habib Dakik

**Affiliations:** ^1^Department of Internal Medicine, American University of Beirut, Beirut, Lebanon; ^2^Department of Diagnostic Radiology, American University of Beirut, Beirut, Lebanon

## Abstract

Congenital abnormalities of the coronary arteries are uncommon but can be associated with important cardiac events depending on their location and the course of the aberrant artery. Conventional angiography has been the gold standard for the diagnosis of these anomalies. The recent development of cardiac CT has allowed accurate and non-invasive depiction of coronary artery anomalies in terms of their origin, course and termination. We describe the case of a patient presenting with acute inferior myocardial infarction who was found to have a very rare congenital abnormality consisting of an anomalous origin of the right coronary artery from the mid-segment of the left anterior descending. Coronary angiography and cardiac CT angiographic images are shown and discussed.

## Case report

**Figure 1.**
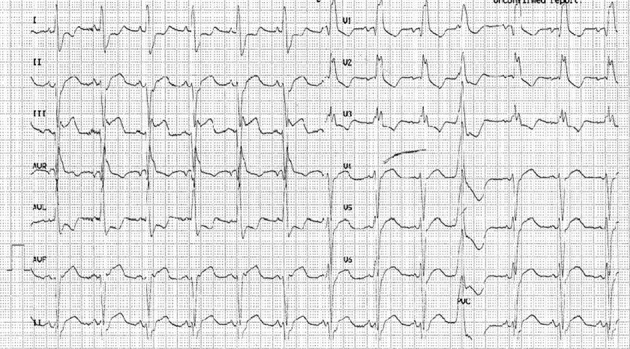
ECG upon presentation to the emergency room showing RBBB and ST segment elevation in leads II, III and AVF.

**Figure 2.**
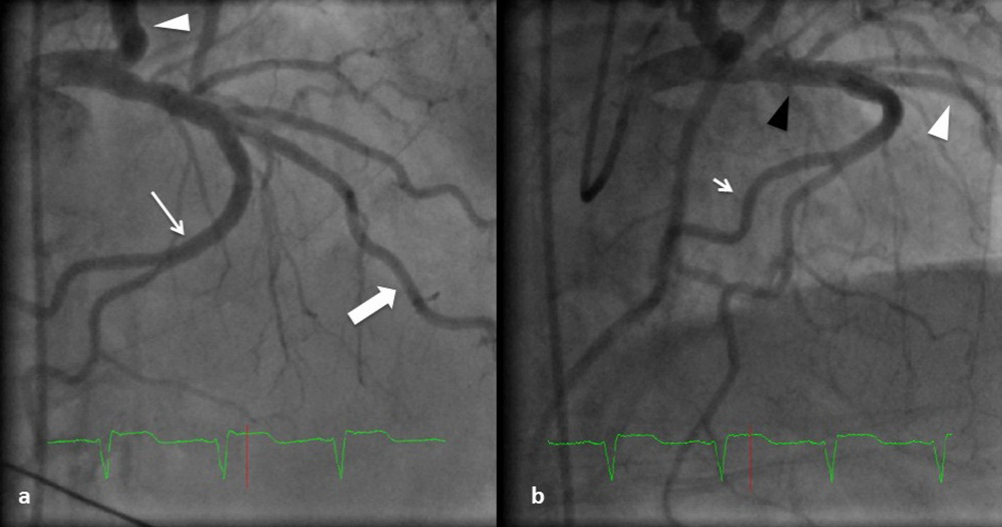
(a) AP cranial view of the left coronary system showing the left main coronary artery, LAD (large arrow), circumflex (arrow head), and the RCA with its aberrant take-off from the mid-LAD segment (small arrow). (b) Right anterior oblique view showing the proximal LAD (black arrow head), distal LAD (white arrow head) and the aberrant RCA taking off from the mid-LAD with its distal branches (small arrow). LAD, left anterior descending; RCA, right coronary artery.

A 65-year-old female with long-standing history of hypertension and Type II diabetes mellitus presented to the emergency department complaining of sudden onset of chest pain and dyspnea. Initial evaluation in the emergency department revealed a BP of 210/120 mmHg, sinus tachycardia at 120 beats min^–1^, and tachypnea. On examination, she had pulmonary oedema with symmetrical and equal pulses in both upper and lower limbs. She was given intravenous furosemide 100 mg, and an electrocardiogram (ECG) was obtained showing sinus rhythm with right bundle branch block (RBBB) and ST segment elevation in leads II, III and AVF (Figure 1). She underwent emergent coronary angiography which showed normal coronary arteries but with aberrant take-off of the right coronary artery (RCA) from the mid-segment of the left anterior descending (LAD) artery (Figure 2). Echocardiography showed inferior wall hypokinesis with an ejection fraction of 50%.

**Figure 3.**
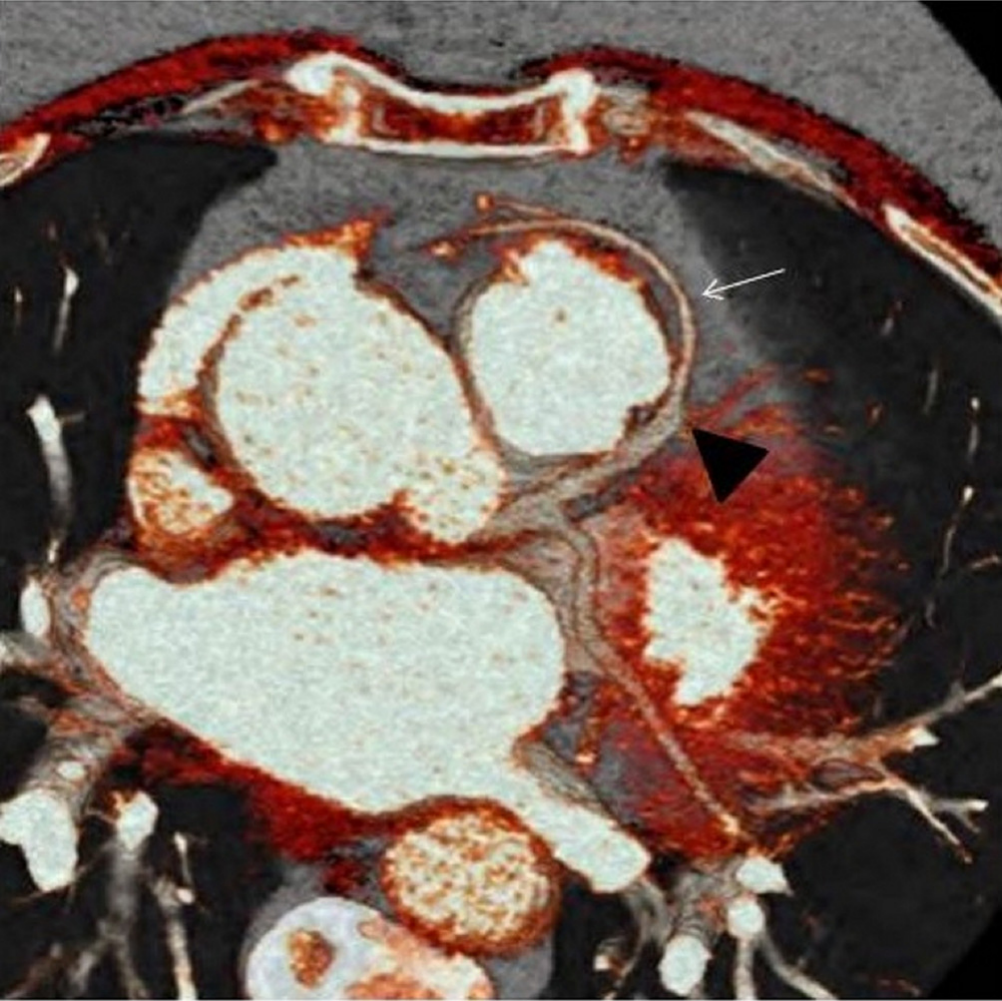
Cardiac CT image showing the left coronary artery and the aberrant RCA (small arrow) with its take-off from the mid-segment of the LAD. LAD, left anterior descending; RCA, right coronary artery.

**Figure 4.**
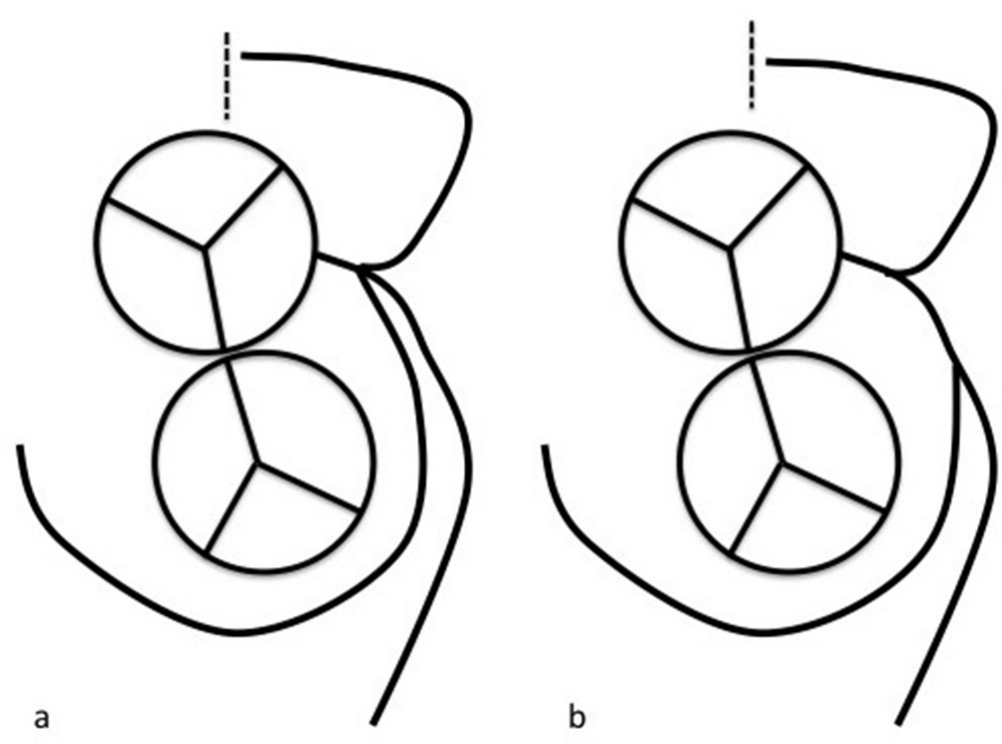
image (a) demonstrates the classical IB1 type of Shirani and Roberts classification where the RCA originates from the distal segment of the left main coronary artery, whereas, image (b) demonstrates the origin of the RCA at the mid-segment of the LAD. LAD, left anterior descending; RCA, right coronary artery.

Patient was admitted to the hospital where her course was stable and her pulmonary oedema resolved. A cardiac CT angiogram (Figure 3) was subsequently performed and it showed an aberrant origin of the RCA from the lateral aspect of the mid-segment of the LAD. The RCA courses circumferentially around the base of the pulmonary trunk and traverses laterally into the atrioventricular groove. The RCA then bifurcates into acute marginal and posterolateral branches. This rare variant has not been categorized in the classification of coronary anomalies; however, it closely resembles the IB1 type of Shirani and Roberts’s classification with the only exception of the RCA origin at the mid-segment and not the proximal segment of the LAD (Figure 4).

## Discussion

Congenital coronary arteries anomalies are uncommon. They are found in about 1.0% of routine cardiac catheterizations.^1^ They vary in type and are classified as anomalies of origin, course and fistulas.^1^ The most commonly described anomaly is separate origin of the LAD and left circumflex from the left sinus of valsalva.^2^ Most cases of coronary arteries anomalies are asymptomatic; however some are associated with increased risk of myocardial infarction and sudden cardiac death.^3^ The mechanism of ischaemia is still unclear and according to prevailing opinion, coronary segments with an anomalous course are no more vulnerable to obstructive atherosclerotic disease than are normal segments in the same individual.^4^

Anomalous origin of the RCA from the LAD artery is a rare occurrence.^5^ Our case demonstrates that this rare anomaly can also be associated with myocardial ischaemia as our patient presented with ST segment elevation myocardial infarction in the RCA territory that was confirmed later by echocardiography showing hypo-kinesis of the inferior wall. The reason for myocardial infarction in our patient is not clear given that the coronary angiogram did not show any significant atherosclerosis. Possible mechanisms include acute angle of take-off and kinking of the RCA as it arises from the LAD, compression of the RCA by the pulmonary trunk or RCA spasm.^6^

## Learning points

1. CT allows accurate and non-invasive depiction of coronary artery anomalies
2. CT coronary angiography can be used as a first-line investigation in the assessment of coronary arteries in patients who have been admitted to the hospital following acute myocardial infarction
3. Coronary artery anomalies can present as acute myocardial infarction or unstable angina. Awareness of this possibility will help in early and correct diagnosis.

## Consent

Written informed consent for the case to be published (including images, case history and data) was obtained from the patient(s) for publication of this case report, including accompanying images.
